# Origin and Evolution of Two Independently Duplicated Genes Encoding UDP- Glucose: Glycoprotein Glucosyltransferases in *Caenorhabditis* and Vertebrates

**DOI:** 10.1534/g3.119.400868

**Published:** 2019-12-03

**Authors:** Diego A. Caraballo, Lucila I. Buzzi, Carlos P. Modenutti, Ana Acosta-Montalvo, Olga A. Castro, María S. Rossi

**Affiliations:** * CONICET-Universidad de Buenos Aires, IFIBYNE, Buenos Aires, Argentina, Universidad de Buenos Aires, Facultad de Ciencias Exactas y Naturales, Departamento de Fisiología, Biología Molecular y Celular, Buenos Aires, Argentina,; † CONICET-Universidad de Buenos Aires, IQUIBICEN, Buenos Aires, Argentina; Universidad de Buenos Aires, Facultad de Ciencias Exactas y Naturales, Departamento de Química Biológica, Buenos Aires, Argentina,; ‡ CONICET-Universidad de Buenos Aires, IQUIBICEN, Buenos Aires, Argentina; Universidad de Buenos Aires, Facultad de Ciencias Exactas y Naturales, Departamento de Química Biológica, Buenos Aires, Argentina, and; § CONICET-Universidad de Buenos Aires, IQUIBICEN, Buenos Aires, Argentina; Universidad de Buenos Aires, Facultad de Ciencias Exactas y Naturales, Departamento de Fisiología y Biología Molecular y Celular, Buenos Aires, Argentina

**Keywords:** UDP- glucose: glycoprotein glucosyltransferase, *Caenorhabditis elegans*, Vertebrates, Purifying selection, Positive selection, Neofunctionalization

## Abstract

UDP- glucose: glycoprotein glucosyltransferase (UGGT) is a protein that operates as the gatekeeper for the endoplasmic reticulum (ER) quality control mechanism of glycoprotein folding. It is known that vertebrates and *Caenorhabditis* genomes harbor two *uggt* gene copies that exhibit differences in their properties.

Bayesian phylogenetic inference based on 195 UGGT and UGGT-like protein sequences of an ample spectrum of eukaryotic species showed that *uggt* genes went through independent duplications in *Caenorhabditis* and vertebrates. In both lineages, the catalytic domain of the duplicated genes was subjected to a strong purifying selective pressure, while the recognition domain was subjected to episodic positive diversifying selection. Selective relaxation in the recognition domain was more pronounced in *Caenorhabditis uggt-b* than in vertebrates *uggt-2*. Structural bioinformatics analysis revealed that *Caenorhabditis* UGGT-b protein lacks essential sequences proposed to be involved in the recognition of unfolded proteins. When we assayed glucosyltrasferase activity of a chimeric protein composed by *Caenorhabditis uggt-b* recognition domain fused to *S. pombe* catalytic domain expressed in yeast, no activity was detected.

The present results support the conservation of the UGGT activity in the catalytic domain and a putative divergent function of the recognition domain for the UGGT2 protein in vertebrates, which would have gone through a specialization process. In *Caenorhabditis*, *uggt-b* evolved under different constraints compared to *uggt-a* which, by means of a putative neofunctionalization process, resulted in a non-redundant paralog. The non-canonical function of *uggt-b* in the worm lineage highlights the need to take precautions before generalizing gene functions in model organisms.

Approximately one-third of all cellular proteins are imported into the lumen of the ER or integrated into its membranes and most of them are glycoproteins. The ER retains some of these proteins while others are exported into the secretory pathway (Wang and Kaufman 2014). The ER uses an elaborate surveillance system called the ER quality control (QC) that monitors the proper folding of these newly synthesized glycoproteins. The QC allows cells to differentiate between native and non-native protein conformations, exporting properly folded proteins to their destination, and eliminating those which fail to fold adequately. Alternatively, misfolded or incompletely formed glycoprotein complexes are translocated to the cytosol where they are finally degraded by proteasomes (Caramelo and Parodi 2007).

The *N*-glycosylation of proteins starts with the addition of a triglucosylated glycan to nascent polypeptide chains by the oligosaccharyltransferase complex. Concomitantly, this triglucosylated glycan is processed by glucosidase I and glucosidase II (GII) (Caramelo and Parodi 2007; Lamriben *et al.* 2016). This trimming process produces monoglucosylated glycoproteins that may interact with two ER-resident lectins, calnexin (CNX) and calreticulin (CRT), which function as non-conventional chaperones (Williams 2006). Monoglucosylated glycans may also be formed by glycan reglucosylation by the UDP- glucose: glycoprotein glucosyltransferase (UGGT) (Caramelo and Parodi 2007). This enzyme is an essential element of the QC because it monitors glycoprotein conformations. UGGT discriminates properly folded from misfolded glycoproteins glucosylating only those which do not display their native conformations (Trombetta *et al.* 1989; Caramelo *et al.* 2003, 2004) Cycles of CNX/CRT-glycoprotein binding and release, catalyzed by the opposing activities of UGGT and GII, persist until glycoproteins attain their native structures or, alternatively, are recognized by cells as irreparably misfolded species or as complexes unable to acquire their full subunit complement, and diverted for their final disposal and degradation (Caramelo and Parodi 2007; Lamriben *et al.* 2016).

UGGTs are monomeric soluble proteins composed of at least two domains (Guerin and Parodi 2003). The N-terminal domain (80% of the sequence) has no homology to other known proteins and is involved in the recognition of misfolded proteins. The C-terminal domain (20% of the sequence) displays a similar size and significant similarity to members of the glucosyltransferase family 8 (Guerin and Parodi 2003). While both structural and experimental evidence supports the idea that the C-terminal domain is the catalytic portion of the enzyme, the role of the N-terminal domain in the recognition of non-native conformers has not been entirely untangled yet. Current evidence suggests that the common determinant recognized is a stretch of hydrophobic residues (Totani *et al.* 2009; Izumi *et al.* 2017) which are exposed in the surface of glycoproteins that present a molten globule, native-close conformation but not random coil or compact native conformations (Caramelo *et al.* 2003, 2004).

Two genes encode UGGT-like proteins in humans (*HUGT1* and *HUGT2*) (Arnold *et al.* 2000), and in *Caenorhabditis*: *Ce-uggt*-a and *Ce-uggt*-b (Buzzi *et al.* 2011). On the contrary, genomes of other model species as *Drosophila melanogaster* (Parker *et al.* 1995), *Arabidopsis thaliana* (Jin *et al.* 2007) and also *Trypanosoma cruzi* (Conte *et al.* 2003) carry a single *uggt* gene. UGGT function is widely conserved across eukaryotes and only a few organisms lack UGGT activity: some protists that make either very short N-linked glycans or no N-linked glycans at all, as *Tetrahymena*, *Giardia* or *Plasmodium* (Banerjee *et al.* 2007; Samuelson and Robbins 2015) and the yeast *Saccharomyces cerevisiae* (Fernández *et al.* 1994; Castro *et al.* 1999). Whereas the lack of UGGT activity is due to a secondary loss of the *uggt* gene in this small group of protists, in *S. cerevisiae* there is a gene that encodes a UGGT-like protein, Kre5p with the same size and subcellular location as canonical UGGTs, but devoid of UGGT activity (Meaden *et al.* 1990).

It has been demonstrated that *Ce*-UGGT-a and *Ce*-UGGT-b have different functions in *C. elegans*. Whereas *Ce*-UGGT-a displayed canonical UGGT activity when it was heterologously expressed in yeast, *Ce*-UGGT-b proved to be completely inactive (Buzzi *et al.* 2011). However, it is unknown if this is because of the divergence of the catalytic or recognition domain, or both. On the other hand, *Ce-uggt*-b is an essential gene; homozygous *Ce-uggt*-b/*Ce-uggt*-b mutant eggs are not able to develop to progressive larval stages even though *Ce*-UGGT-a is fully active (Buzzi *et al.* 2011), while *Ce-uggt-a(RNAi)* worms only show subtle deleterious phenotypes as those found in mutant worms that lack both CNX and/or CRT (Park *et al.* 2001; Lee *et al.* 2005). On the other hand, several reports show conflicting evidence about HUGT2 activity. Arnold and co-workers determined that HUGT2 expressed in mammalian cells was inactive but its C-terminal catalytic domain was still functional (Arnold and Kaufman 2003). On the other hand, Takeda and collaborators expressed several truncated and chimeric proteins combining different regions of HUGT1 and HUGT2 and showed that both HUGT1 and HUGT2 and even N-truncated proteins comprising only the C-terminal domain, were able to glucosylate synthetic substrates (Takeda *et al.* 2014). In addition, both mouse UGGT1 and UGGT2 displayed activity in hybridoma cells in which isoform-specific knockdowns were performed (Prados *et al.* 2013). Although the currently available evidence about vertebrate UGGT2 cannot be put together easily, these results would support the notion that both vertebrate UGGTs are active.

This scenario led us to think about different evolutionary pathways of *uggt* genes in *C. elegans* and vertebrates. In the present work, we investigate the origin of these genes in a broad phylogenetic framework and estimate modes of molecular evolution acting on their sequences in vertebrates and *Caenorhabditis* worms, the only eukaryotic lineages known to harbor two *uggt* genes.

## Materials and Methods

### Bioinformatic procedure, phylogeny and selection tests

#### Sequence retrieval and alignment:

We analyzed a total of 195 UGGT (and UGGT-like) protein sequences retrieved from Genbank (Clark *et al.* 2016) and Wormbase (“Wormbase”) databases, representing all major eukaryotic groups (Additional files 1 and 2). UGGT sequences from plants, fungi, protozoans, heterokonts, and bilateral animals were included. The sequences belonging to bilateral animals were retrieved from Genbank based on the UGGT tree (ENSGT00390000004600) present in the ENSEMBL database (“Ensemble”). Truncated sequences were excluded from the analysis. Within vertebrates, some species showed only one of the two paralogs (or the paralog was a partial and or/low-quality sequence), and both copies were excluded from the analysis. As a result, we retained 55 sequences of each paralog in both phylogenetic and selection analyses. In the case of *S. cerevisiae*, we included the sequence of *KRE5* which is the UGGT homolog. Protein sequences were aligned using Clustal Omega (Additional File 3) (“Clustal O”).

A total of 47 UGGT nematode sequences were retrieved using BioMart via the Wormbase ParaSite database (https://parasite.wormbase.org). To get this final set of sequences we initially retrieved the list of all nematodes orthologs of uggt-a and uggt-b from Wormbase. Using the Wormbase stable IDs we retrieved orthologous coding sequences with BioMart. Duplicate sequences and those that did not start with a Methionine were excluded from the final set. The sequences of two highly related rhabditid genera, *Oscheius* and *Diploscapter* were included to test the monophyly of *Caenorhabditis* UGGTs. As occurred with *Caenorhabditis* sequences, we retrieved almost identical sequences in *Diploscapter coronatus* which belonged to different scaffolds that do not represent a duplication event, but rather represent intra-individual (haplo-genomic) variation of a diploid genome (we kept the sequence DCO_002639, Scaffold scf7180000986577).

Within *Caenorhabditis*, we included 7 *uggt-a* and *uggt-b* sequences from the species: *C. elegans*, *C. inopinata*, *C. japonica*, *C. sinica*, *C. brenneri*, *C. briggsae*, and *C. remanei*. We retrieved four *C. angaria* uggt sequences, all of them representing partial *uggt* sequences in the same location, and for this reason, they were excluded from the analysis. We retrieved 3 *uggt* gene sequences of *C. latens*, but again were incomplete and thus were excluded from the analysis. In *C. nigoni* we retrieved a complete *uggt-a* sequence, and a truncated *uggt-b*, so we decided to exclude the pair from the final analysis. In *C. tropicalis* we found a complete *uggt*-a sequence, but no *uggt-b* ortholog, so we excluded it from the final analysis. In these two last species, we tested the phylogenetic position of *uggt-a* and it resulted included within the *uggt-a* clade of all *Caenorhabditis* (not shown) but were excluded from the phylogeny and selection analysis since we decided to compare duplicated copies.

Phylogenetic analyses were run in MrBayes 3.2.6 (Ronquist *et al.* 2012) on the CIPRES Science Gateway (Miller *et al.* 2015). A first analysis was run sampling across fixed amino acid rate matrices, where the Jones model depicted the highest support. Since *uggt-a* and *uggt-b* resulted reciprocally monophyletic but together did not form a monophyletic group, we ran nucleotide and protein phylogenies to test their relationships within nematodes, confirming *uggt-a* and *uggt-b* are paraphyletic as described in Results section.

We then ran the definitive analysis fixing the Jones model for 1.3x10^7^ Markov Chain Monte Carlo (MCMC) generations, sampling every 1000 generations. A suitable burnin fraction was selected based on the resulting Estimated Sample Size (ESS) which was below 0.01 by 21.65% of the MCMC chain, where it showed as well a stationary trace distribution. The UGGT sequence of the grass species *Oryza brachyantha* was set as outgroup.

#### Conservation analysis:

Prior to selection analysis, we screened for conservation at the protein level. To this end, we performed alignments of each UGGT separately for the two clades where UGGT went through duplication: vertebrates and *Caenorhabditis*. Gap rich positions were removed with GBlocks 0.91b (Castresana 2000) using the “with half” option (positions with a gap in less than 50% of the sequences are kept). Conservation was inspected using PlotCon (“Plotcon”), using the EBLOSUM62 amino acid similarity matrix, with a window size of 200 to account for conservation in neighboring sites. Corrected distances between both UGGTs from *H. sapiens* and *C. elegans* were computed with MEGA version 6 (Tamura *et al.* 2013) using the Jones-Taylor-Thorton model.

#### Selection analysis:

To assess if natural selection affected the evolution of UGGTs of vertebrates and *Caenorhabditis*, we employed codon-based and lineage-based Bayesian and maximum likelihood approaches to estimate rates of non-synonymous (dN) to synonymous substitutions (dS). To this end, we performed protein alignments for each separate UGGT of vertebrates and *Caenorhabditis*. Separate alignments were performed for each protein domain. Regions spanning UGGT recognition and catalytic domains were retrieved from the NCBI’s conserved domain database (Marchler-Bauer *et al.* 2017), taking as references HUGT1 and HUGT2 sequences in the vertebrates alignments. The *Ce*-UGGT-a sequence was taken as reference in the *Caenorhabditis* alignment, whereas domains of *Ce*-UGGT-b were inferred from their relative positions to *Ce*-UGGT-a. Additional file 4 shows the amino acid positions corresponding to each UGGT domain taken as reference sequences. The resulting protein alignments were used as references for converting nucleotide alignments into codon alignments employing PAL2NAL (Suyama *et al.* 2006), and gap-rich positions were removed using GBlocks as described above.

The unrooted vertebrate and *Caenorhabditis* UGGTs subtrees were uploaded to the Datamonkey webserver (Kosakovsky Pond and Frost 2005; Delport *et al.* 2010) and selection was inferred using the following codon-based methods. Single sites under selection were identified using Single Likelihood Ancestral Counting (SLAC) (Kosakovsky Pond and Frost 2005), Fixed Effects Likelihood (FEL) (Kosakovsky Pond and Frost 2005), Internal Fixed Effects Likelihood (IFEL) (Kosakovsky Pond *et al.* 2006), Random Effects Likelihood (REL) (Kosakovsky Pond and Frost 2005), Mixed Effects Model of Evolution (MEME) (Murrell *et al.* 2012), as well as Fast Unconstrained Bayesian AppRoximation (FUBAR) (Murrell *et al.* 2013). SLAC infers dN and dS at each codon position comparing observed and expected rates based on a single ancestral sequence reconstruction. FEL estimates and compares dN and dS independently on a per-site basis. IFEL performs the same analysis as FEL except that selection is only tested along internal branches of the phylogeny. REL performs also a per-site dN and dS estimation but allows for overall dN/dS (ω) heterogeneity. MEME aims to detect single sites evolving under positive selection along particular branches. FUBAR enables larger numbers of site classes and identifies positively selected sites using a Bayesian framework. Significance thresholds for selection tests were *P* ≤ 0.10 for SLAC, FEL, IFEL and MEME, posterior probability ≥0.90 for FUBAR and Bayes factor ≥50 for REL.

Selection at each domain was also tested using tree-based methods at the Datamonkey server: RELAX and BUSTED. Given two subsets of branches in a phylogeny, RELAX (Wertheim *et al.* 2015) determines whether selective strength was relaxed or intensified in one of these subsets relative to the other. BUSTED (Branch-Site Unrestricted Statistical Test for Episodic Diversification, (Murrell *et al.* 2015) tests for evidence of Episodic Diversifying Selection (EDS) in at least one site and one branch of the phylogeny.

### Experimental procedures

#### Media, strains and reagents:

*gpt1/alg6 S. pombe* (Sp61G4A (h-, *ade6-M210*, *ade1*, *leu1-32*, *ura4*-D18, *gpt1*::*ura4*-D1684, *alg6*::*ura4+*) was used for heterologous expression (Fanchiotti *et al.* 1998). *S. pombe* cells were grown at 28° in YEA medium or MM medium supplemented with adenine or leucine as needed (Moreno *et al.* 1991). *Escherichia coli* strain STBL3 (Invitrogen, Carlsbad, CA) was grown in LB medium with 100 µg/ml ampicillin when needed. Reagents for yeast media were obtained from Difco Laboratories (Detroit, MI). N-Methyl-1-deoxynojirimycin (NMDNJ) was from Research Chemicals (North York, ON, Canada). Enzymes used for DNA procedures were from New England Biolabs (Ipswich, MA), KOD Hot Start DNA Polymerase was from Merck (Darmstadt, Alemania) andpCR2.1-TOPO Vector was from Invitrogen (Carlsbad, CA). Unless otherwise stated, all other reagents were from Sigma (St. Louis, MO). UDP-[^14^C]Glc was synthesized as previously reported with slight modifications (Wright A 1965). Protein concentrations were determined by Bio-Rad Protein Assay as described by the manufacturer.

#### Cloning of c-myc labeled Ce-uggt-a, Ce-uggt-b, and chimeric proteins:

C-terminally *c-myc* labeled *Ce-uggt-a* and *Ce-uggt-b* optimized versions were synthesized using pREP3X-*uggt-a* and pREP3X-*uggt-b* as template (Buzzi *et al.* 2011), using oligonucleotide primers GTAF and GTAMYCR for *uggt-a* and GTBF and GTBMYCR for *uggt-b* and KOD Hot Start DNA Polymerase. PCR products were first cloned into the pCR2.1-TOPO vector and then introduced into the XhoI and BamHI sites of pREP3X. The c-*myc* sequence was inserted immediately before the ER retrieval sequence in both constructions. These plasmids were named pREP3X-*uggt-a- c-myc* and pREP3X-*uggt-b- c-myc* respectively. The plasmid encoding the c-terminally *c-myc* labeled *S. pombe gpt1+* was already available (pREP3X-*gpt1+-c-myc*) (Guerin and Parodi 2003) (constructions 3, 4 and 2, in that order, Figure 6A). Expression plasmids codifying for c-terminally *c-myc* labeled versions of two chimeric proteins composed by the N-terminal domain of *Ce*-UGGT-a (amino acids 1 to 1200) fused to the C-terminal domain of *Sp*UGGT (amino acids 1155 to 1447) named chimera I, and by the N-terminal domain of *Ce*-UGGT-b (amino acids 1 to1093) fused to the C-terminal domain of *Sp*UGGT (amino acids 1155 to 1447) named chimera II (constructions 5 and 6, Figure 6A), were synthesized using the overlapping PCR procedure (Bryksin and Matsumura 2010). First, PCR fragments encoding the N-terminal and C-terminal domains of the chimeric proteins were amplified using the templates and primers described in Additional File 5, Table S1. A second PCR using as templates pairs of appropriate PCR fragments obtained in the first PCR amplification and primers containing 20-30 base pairs of sequence overlap with the specific sequences at the end each of the two PCR fragments were performed to obtain the full length DNA sequences encoding chimeras I and II (Figure 6A) as indicated in Additional File 5 Table S2. The complete sequences were cloned into pCR2.1-TOPO vector and in a second step inserted into the XhoI and BamHI sites of pREP3X vector to produce c-terminally *c-myc* labeled expression plasmids pREP3X-chimera I, pREP3X-chimera II. Primer sequences used in these constructions are described in Additional File 5, Table S3.

#### Chimeric protein expression in gpt1/alg6 double mutant S. pombe cells:

Expression plasmids were electroporated into *gpt1/alg6S. pombe* cells and transformants were selected on MM plates plus adenine containing 15 mM thiamine. To test the accurate expression of the different proteins, 200 µg of *S. pombe* microsomal proteins were analyzed in 8% SDS-PAGE and subjected to Western blot analysis using an anti-*c-myc* antibody (Sigma) and a commercial *ECL Plus Western Blotting* chemiluminescence kit (Thermo Scientific *Pierce*).

#### UGGT assay:

UGGT activity was measured using UDP-[^14^C]Glc as a sugar donor and denatured thyroglobulin as a glucosyl acceptor as previously described (Trombetta *et al.* 1989). Briefly, the incubation mixtures contained, in a total volume of 50 µl, 0.2 µl of 8 M urea denatured bovine thyroglobulin, 10 mM CaCl_2_, 3 µ Ci UDP-[^14^C]Glc, 0.4% Lubrol, 1 mM NMDNJ was from Research Chemicals (North York, ON, Canada), and 300 µg of yeast microsomal protein. Reactions were stopped by the addition of 1 ml of 10% trichloroacetic acid. After centrifugation, the pellets were twice washed with 1 ml of 10% trichloroacetic acid and counted. *Schizosaccharomyces pombe* microsomes were prepared as already described (Trombetta and Parodi 1992; Fernández *et al.* 1994).

#### Structural bioinformatics analysis:

UGGT-a and UGGT-b Homology Modeling. The *Ce*-UGGT-a and *Ce*-UGGT-b sequences were sourced from the UniProt server and aligned to the *C. thermophilus* UGGT using the Clustal Omega server (“Clustal O”) and structure mapping over sequence alignment were performed with Espript web server (Robert X 2014). Using this alignment and the CtUGGT crystal structure, homology models for *Ce*-UGGT-a and *Ce*-UGGT-b were built using Modeler (Webb, B. Sali 2016). Structural comparison and images were produced using VMD software (Humphrey, W., Dalke, A. and Schulten 1996).

### Data availability

All supplementary/additional files are available in the GSA figshare portal.

Supplementary Figure 1 shows branch-specific relaxation of UGGT in *Caenorhabditis*. Supplementary Figure 2 shows branch-specific relaxation of UGGT in Vertebrates. Supplementary Figure 3 shows Western-blot analysis of the expression of the c-Myc labeled full length and chimeric proteins.

Table S1 lists templates and primers used for amplification of UGGT N- and C-terminal domains of S. pombe UGGT and *Ce*-UGGT-a and *Ce*-UGGT-b.

Table S2 lists templates and primers used in the PCR amplification of the full-length fragments encoding chimeric UGGTs.

Table S3 shows DNA primer sequences used in this work.

Additional File 1 lists all sequences and accession numbers used in phylogenetic/selection analysis.

Additional File 2 contains the unaligned set of 195 UGGT (and UGGT-like) protein sequences used in phylogenetic inference.

Additional File 3 contains the aligned set of 195 UGGT (and UGGT-like) protein sequences used in phylogenetic inference.

Additional File 4 shows aminoacidic positions corresponding to each UGGT domain taken as reference sequences.

Additional File 5 contains Table S1, Table S2, Table S3 and Supplementary Figure S3.

The interactive version of the phylogenetic tree has been uploaded to the iTOL server (https://itol.embl.de/tree/181461382483101567002659). Supplemental material available at figshare: https://doi.org/10.25387/g3.11234654.

## Results

### Independent duplications of uggt genes in Caenorhabditis and vertebrates

**Figure 1:**
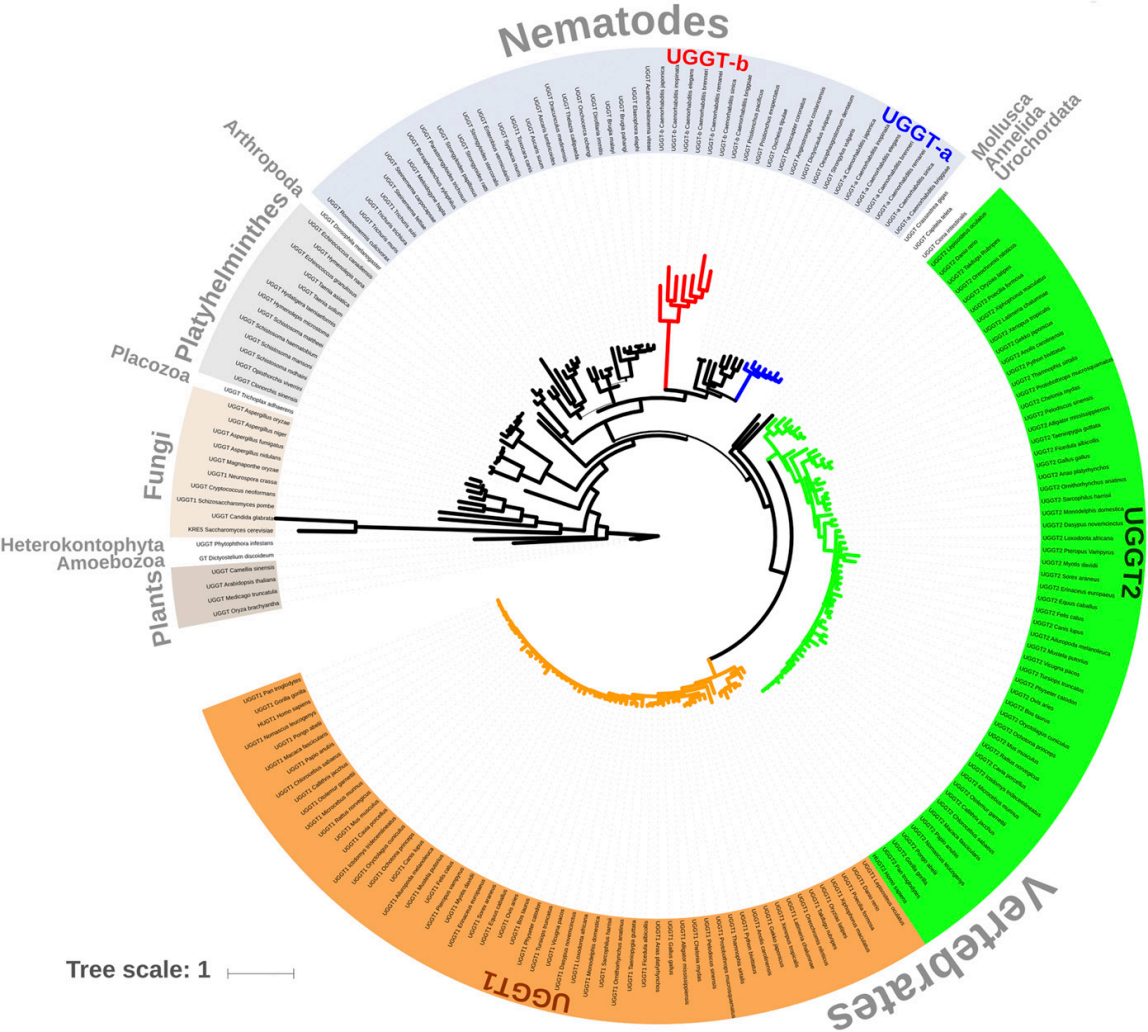
Bayesian phylogenetic tree based on 195 UGGT (and UGGT-like) protein sequences, representing all major eukaryotic groups. Branch thickness is proportional to node posterior probability. Branches derived from nodes with posterior probability values under 0.7 are shown in light gray. The UGGT sequence of the grass species *Oryza brachyantha* was set as outgroup. UGGTs of vertebrates and *Caenorhabditis* diverged in distant groups, in chordates and nematodes respectively, reflecting that independent duplication events occurred in the ancestor of these groups. The interactive version of the tree has been uploaded to the iTOL server (link). The scale bar represents 1 amino acidic substitution per site.

Phylogenetic relationships resulting from the Bayesian analysis based on 195 UGGT and UGGT-like sequences from all major eukaryotic groups reveal that *uggt* genes went through independent duplications in vertebrates and *Caenorhabditis* (Figure 1). Within vertebrates, UGGT1 and UGGT2 diverged from the basal node as two reciprocally monophyletic groups that compose in turn a monophyletic group, reflecting that the duplication event occurred in the ancestor of vertebrates and both gene copies were maintained throughout the evolution of this lineage. In the case of *Caenorhabditis*, UGGT-a and UGGT-b are closely related reciprocal monophyletic groups but are paraphyletic when taken together (Figure 1). A set of non-duplicated rhabditid UGGTs which depicts more phylogenetic affinity with UGGT-a, in terms of branch length and topology, interposes both *Caenorhabditis* subclades. The topologies of both subtrees, based on UGGT1/UGGT-a or UGGT2/UGGT-b, are congruent with the phylogeny of vertebrates (Fong *et al.* 2012) and *Caenorhabditis* species (Kiontke *et al.* 2011; Stevens *et al.* 2019).

Branch lengths in the *Ce*-UGGT-b subtree are remarkably larger than those of *Ce*-UGGT-a (Figure 1), suggesting that this protein may have experienced a relaxation in purifying selection and/or a process of diversifying (positive) selection. In contrast, both vertebrate UGGTs show comparable branch lengths (Figure 1). Table 1 shows corrected pairwise distances (and standard deviation) between both *Homo sapiens* and *C. elegans* UGGTs amino acid sequences. Distance values between HUGT1 and HUGT2 (0.445) are lower than that of *Ce*-UGGTs (0.560). In turn, distances between any of the *C. elegans* and human HUGTs are comparable to the distance between the two copies of the worm species.

**Table 1:** Pairwise distances between HUGTs and *Ce*-UGGTs

		*C. elegans*		*H. sapiens*	
		*Ce*-UGGT-a	*Ce*-UGGT-b	HUGT1	HUGT2
*C. elegans*	*Ce*-UGGT-a	—			
	*Ce*-UGGT-b	0.560 (0.014)	—		
*H. sapiens*	HUGT1	0.551 (0.012)	0.539 (0.024)	—	
	HUGT2	0.538 (0.016)	0.544 (0.021)	0.445 (0.012)	—

Corrected pairwise distances (and standard deviation) between *Homo sapiens* and *Caenorhabditis elegans* UGGTs aminoacidic sequences, under the Jones-Taylor-Thorton model.

### Variability in recognition and catalytic domains

**Figure 2:**
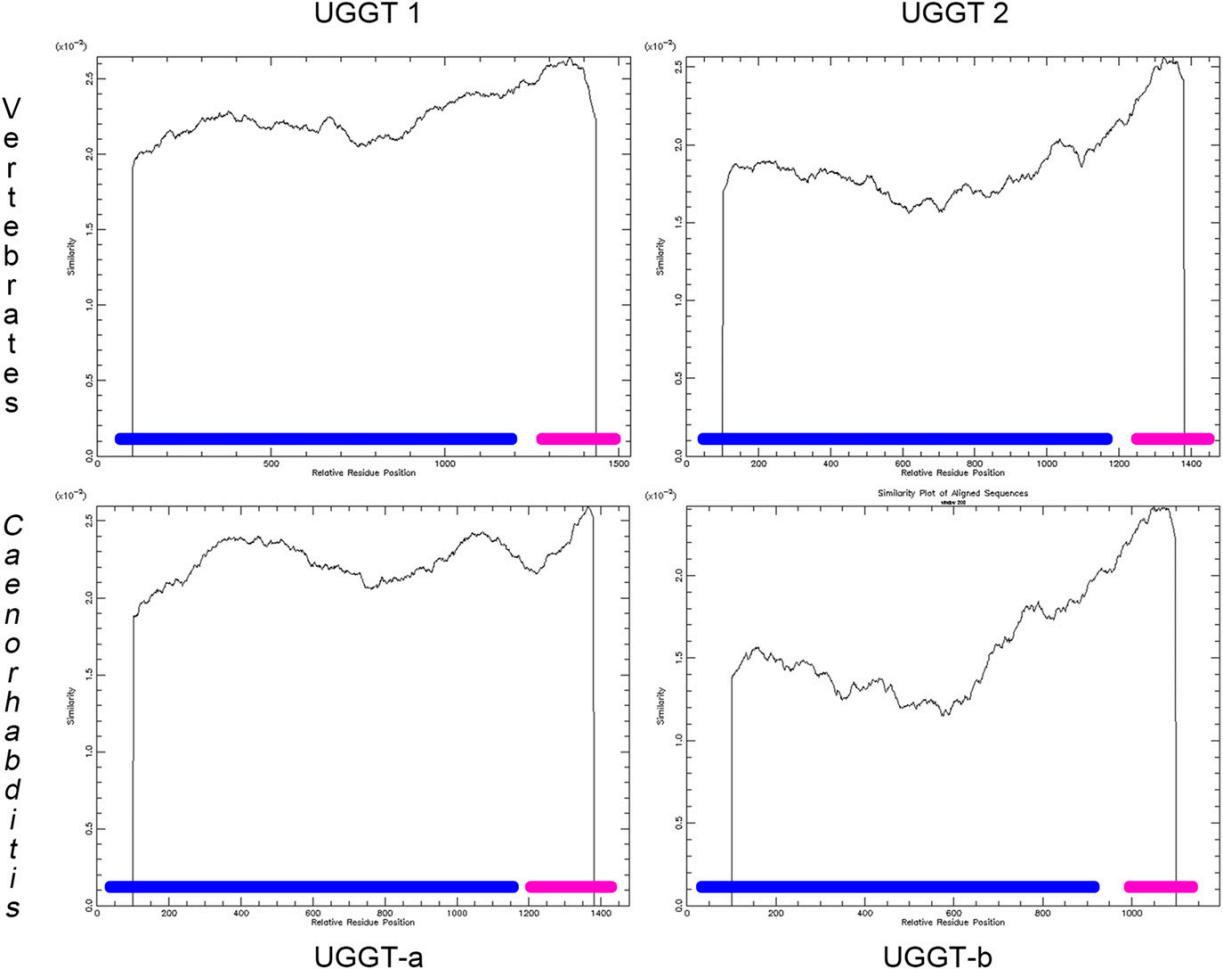
Conservation plot showing the average similarity score at individual amino acid positions from multiple sequence alignments of UGGTs from vertebrates and *Caenorhabditis*. The plots were generated with EMBOSS PlotCon using the EBLOSUM62 comparison matrix and a window size of 200. The recognition and catalytic domains are denoted with blue and purple bars, respectively.

Direct inspection of protein multiple sequence alignments and conservation plots were performed for vertebrate and *Caenorhabditis* UGGTs (Figure 2). In both lineages, UGGT1/UGGT-a amino acid sequences show higher levels of conservation than those of UGGT2/UGGT-b, but the difference between both genes is much more substantial in the worm clade. In addition, there is a difference in sequence conservation between catalytic and recognition domains (Figure 2). The catalytic domain is conserved between both UGGTs, in vertebrates and *Caenorhabditis* (Figure 2), while the recognition domain depicts higher variability levels than those found in the former.

### Positive and purifying selection in recognition and catalytic domains

**Figure 3:**
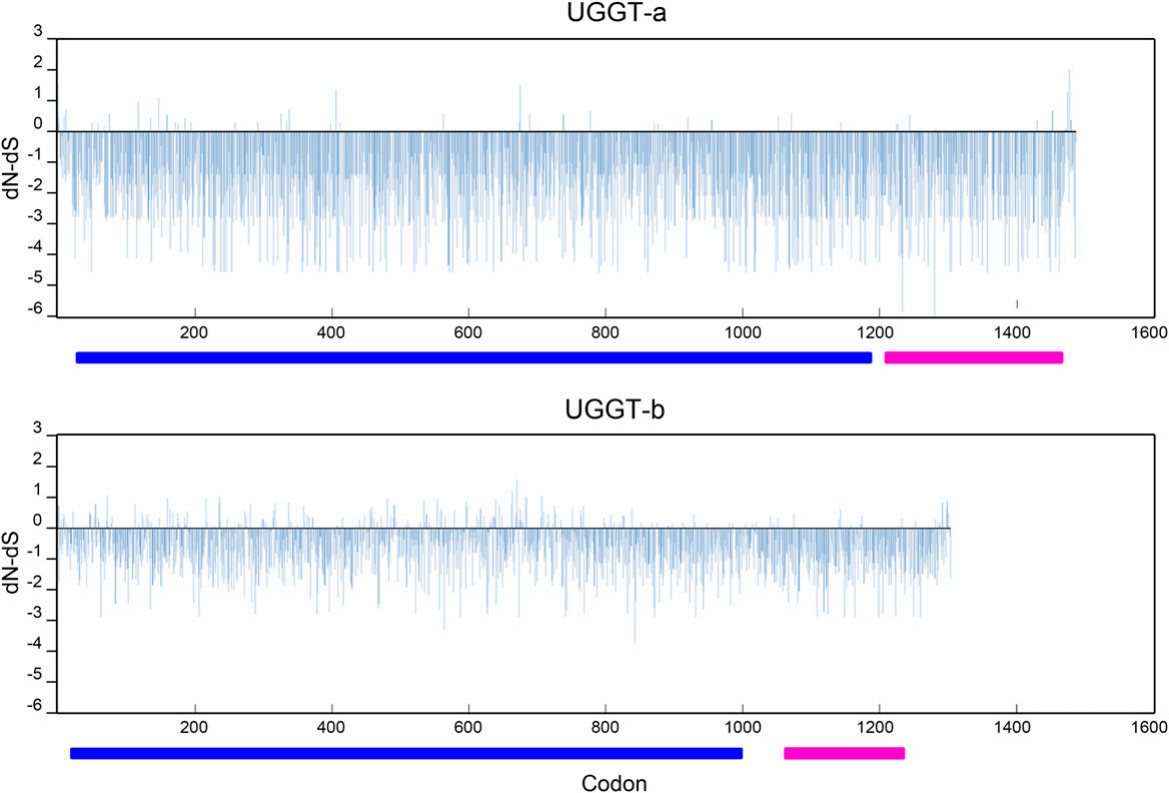
Estimated dN/dS ratios for UGGT codons in *Caenorhabditis*. Codon-based analysis for UGGT selection was performed by multiple methods; shown here are results from the SLAC method. The recognition and catalytic domains are denoted with blue and purple bars, respectively.

**Figure 4:**
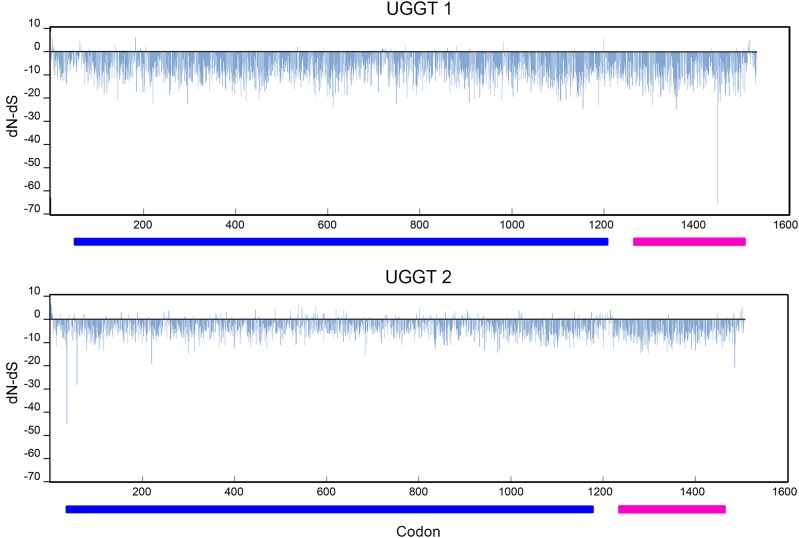
Estimated dN/dS ratios for UGGT codons in vertebrates. Codon-based analysis for UGGT selection was performed by multiple methods; shown here are results from the SLAC method. The recognition and catalytic domains are denoted with blue and purple bars, respectively

Estimates of the ratio of non-synonymous *vs.* synonymous substitutions (dN/dS) of recognition and catalytic domains revealed that negative selection prevailed over positive selection in both regions of UGGTs in vertebrates as well as in *Caenorhabditis* (Figures 3 and 4). Within *Caenorhabditis*, codon-based methods showed a predominant proportion of negatively selected codons in both domains, although this preponderance is almost absolute in the catalytic domain, which in turn shows few/no codons under positive selection (Figure 3, Table 2). In both domains, UGGT-b depicted a higher number of sites under positive selection while a markedly smaller proportion of sites under purifying selection in the recognition domain. Although less pronounced, an analogous pattern was observed in vertebrates (Figure 4, Table 3). In both domains, UGGT2 showed a higher number of positively selected sites in comparison with UGGT1, and a lower proportion of sites under purifying selection.

**Table 2:** Codon-based analysis of selection in *Caenorhabditis* UGGTs

Protein	Sequences	Domain	Type of selection	SLAC	FEL	IFEL	REL	MEME	FUBAR	By at least one method	Relative to sequence length
				(0.1)	(0.1)	(0.1)	(50)	(0.1)	(0.9)		
UGGT-a	7	Recognition	**Positive**	0	0	3	4	6	0	11	1%
		(1146 codons)	**Negative**	502	796	318	796	—	983	1020	89%
		Catalytic	**Positive**	0	0	0	0*^a^*	0	0	0	0%
		(246 codons)	**Negative**	110	175	33	246*^a^*	—	180	187	76%
UGGT-b	7	Recognition	**Positive**	1	8	12	2	29	2	42	4%
		(982 codons)	**Negative**	275	429	192	349	—	586	622	63%
		Catalytic	**Positive**	0	0	0	0*^a^*	2	0	2	1%
		(248 codons)	**Negative**	104	176	74	248*^a^*	—	188	196	79%

Results of multiple codon-based analysis of selection in *Caenorhabditis* UGGT-a and UGGT-b. Significant positively and negatively selected sites detected by SLAC, FEL, IFEL, REL, MEME and FUBAR are shown. Significance thresholds are indicated between parentheses, corresponding to p-values (SLAC, FEL, IFEL and MEME), posterior probability (FUBAR) and Bayes Factor (REL).

a No rates with dN > dS were inferred for this datasets, suggesting that all sites are under purifying selection.

**Table 3:** Codon-based analysis of selection in vertebrate UGGTs

Protein	Sequences	Domain	Type of selection	SLAC	FEL	IFEL	REL	MEME	FUBAR	By at least one method	Relative to sequence length
				(0.1)	(0.1)	(0.1)	(50)	(0.1)	(0.9)		
UGGT1	55	Recognition	**Positive**	0	2	0	7	48	0	52	4%
		(1157 codons)	**Negative**	938	968	888	859	—	1071	1071	93%
		Catalytic	**Positive**	0	0	0	0	3	0	3	1%
		(248 codons)	**Negative**	216	221	216	0	—	242	242	98%
UGGT2	55	Recognition	**Positive**	4	8	9	4	98	1	100	9%
		(1154 codons)	**Negative**	693	774	682	749	—	948	948	82%
		Catalytic	**Positive**	0	0	0	0	9	0	9	4%
		(248 codons)	**Negative**	208	213	210	0	—	232	232	94%

Results of multiple codon-based analysis of selection in vertebrate UGGT1 and UGGT2. Significant positively and negatively selected sites detected by SLAC, FEL, IFEL, REL, MEME and FUBAR are shown. Significance thresholds are indicated between parentheses, corresponding to p-values (SLAC, FEL, IFEL and MEME), posterior probability (FUBAR) and Bayes Factor (REL).

Lineage-based methods were applied to analyze evidence of relaxation or Episodic Diversifying Selection (EDS) in vertebrates and *Caenorhabditis* separately, setting the UGGT2/UGGT-b clade as foreground (test) and UGGT1/UGGT-a as background (or reference). The Relax test revealed significant relaxation of purifying selection in the recognition domain of the *Caenorhabditis* UGGT-b (Table 4). In contrast, the test for selection relaxation was not significant for the catalytic domain in UGGT-b compared to UGGT-a (Table 4). This is due to the presence of a highly differentiated segment of the UGGT-a sequence of *C. sinica*, which is part of the reference sequences (see Materials and Methods), that may be obscuring selection relaxation of UGGT-b in relation to UGGT-a. Indeed, when excluding both *C. sinica* UGGTs from the analysis, significant relaxation in the catalytic domain of UGGT-b is corroborated (k = 0.27, *P* = 0.00). Different purifying selection intensities are observed along UGGT-b branches, which depict a more relaxed pattern of evolution in the recognition domain, while the catalytic domain shows a generalized pattern of negative selection intensification, but as mentioned this pattern is reverted when excluding *C. sinica* (Supplementary Figure 1). In line with this result, evidence of EDS was found in the recognition domain, but not in the catalytic one as revealed by the BUSTED test (Table 4), even when excluding *C. sinica* UGGTs (*P* = 0.622).

**Table 4:** Relaxation and Episodic Diversifying Selection in UGGTs

	Relax		BUSTED	
	Recognition domain	Catalytic domain	Recognition domain	Catalytic domain
**Vertebrates**	Relaxation	Relaxation	EDS	No EDS
	k = 0.36 (*P* = 0.00)	k = 0.69 (*P* = 2.52 e^-8^)	LRT p-value = 0.00	LRT p-value = 0.35
***Caenorhabditis***	Relaxation	No relaxation*^a^*	EDS	No 2EDS*^b^*
	k = 0.43 (*P* = 0.00)	k = 0.22 (*P* = 1.00)	LRT p-value = 0.00	LRT p-value= 0.61

Analysis of evidence of relaxation (Relax) or Episodic Diversifying Selection (BUSTED) in vertebrates and *Caenorhabditis*, setting the UGGT2/UGGT-a clade as foreground (test) and UGGT1/UGGT-b as background (or reference). In the Relax test, k denotes the selection intensity parameter. A significant result of k > 1 indicates that selection strength has been intensified along the test branches, and a significant result of k < 1 indicates that selection strength has been relaxed along the test branches.

a when excluding both *C. sinica* UGGTs from the analysis, significant relaxation in the catalytic domain of UGGT b is corroborated (k = 0.27, *P* = 0.00).

b No evidence of EDS is found when excluding *C. sinica* UGGTs (*P* = 0.622).

Similar results were obtained among vertebrate UGGT2, where significant relaxation was found in both domains and evidence of EDS only in the recognition domain (Table 4). While there is a predominant relaxation along branches of the recognition domain, a strong negative selective pressure prevailed in the catalytic domain (Supplementary Figure 2).

Taken together, these results reflect a generalized background of purifying selection in all UGGTs, suggesting that both duplicates, in vertebrates as well as in *Caenorhabditis*, have been subjected to functional constraints. The catalytic domain is highly conserved as a product of a strong negative selective pressure and shows almost no evidence of positive selection. In contrast, the recognition domain has undergone a positive diversifying selection process in UGGT2/UGGT-b of both lineages. Purifying selective pressure relaxation in the UGGT-b reached higher magnitudes in *Caenorhabditis* compared to vertebrate UGGT2, especially in its recognition domain (Table 4, excluding *C. sinica* when comparing catalytic domains), which is in agreement with the higher overall divergence of this paralog in the worm genus.

### The N-terminal recognition domain of UGGT-b is unable to bind unfolded proteins

It has been previously shown that UGGT-b lacked canonical UGGT activity when it was expressed in *alg6gpt1*- double mutant *S. pombe* cells, which lack UGGT and transfer Man_9_-GlcNAc_2_ instead of the complete glycan, while UGGT-a was fully active (Buzzi *et al.* 2011). There are two plausible explanations for the lack of UGGT-b activity: that either the N-terminal domain or the C-terminal catalytic domain have lost their activities and alternatively, that both domains would have done so. Roversi and collaborators have examined amino acid sequences of C-terminal domains from more than a dozen of UGGTs (including UGGT-a and UGGT-b) and compared them with that of two structurally well-characterized homologous glycosyltransferases (*Neisseria meningitidis* galactosyltransferase and *Anaerococcus prevotii* glycosyltransferase) (Roversi *et al.* 2017). This analysis showed that C-terminal amino acid sequences of both UGGT-a and UGGT-b were highly conserved and that they are extremely similar to that of the homologous glycosyltransferases. In particular, all the amino acids implicated in the catalysis, those that participate in the interaction with divalent-metals and UDP-Glucose as well as those assumed to be involved in substrate binding were conserved both in UGGT-a and UGGT-b (S1 Appendix, [29]). All this evidence strongly suggests that the UGGT-b C-terminal domain has retained its glycosyltransferase activity.

**Figure 5:**
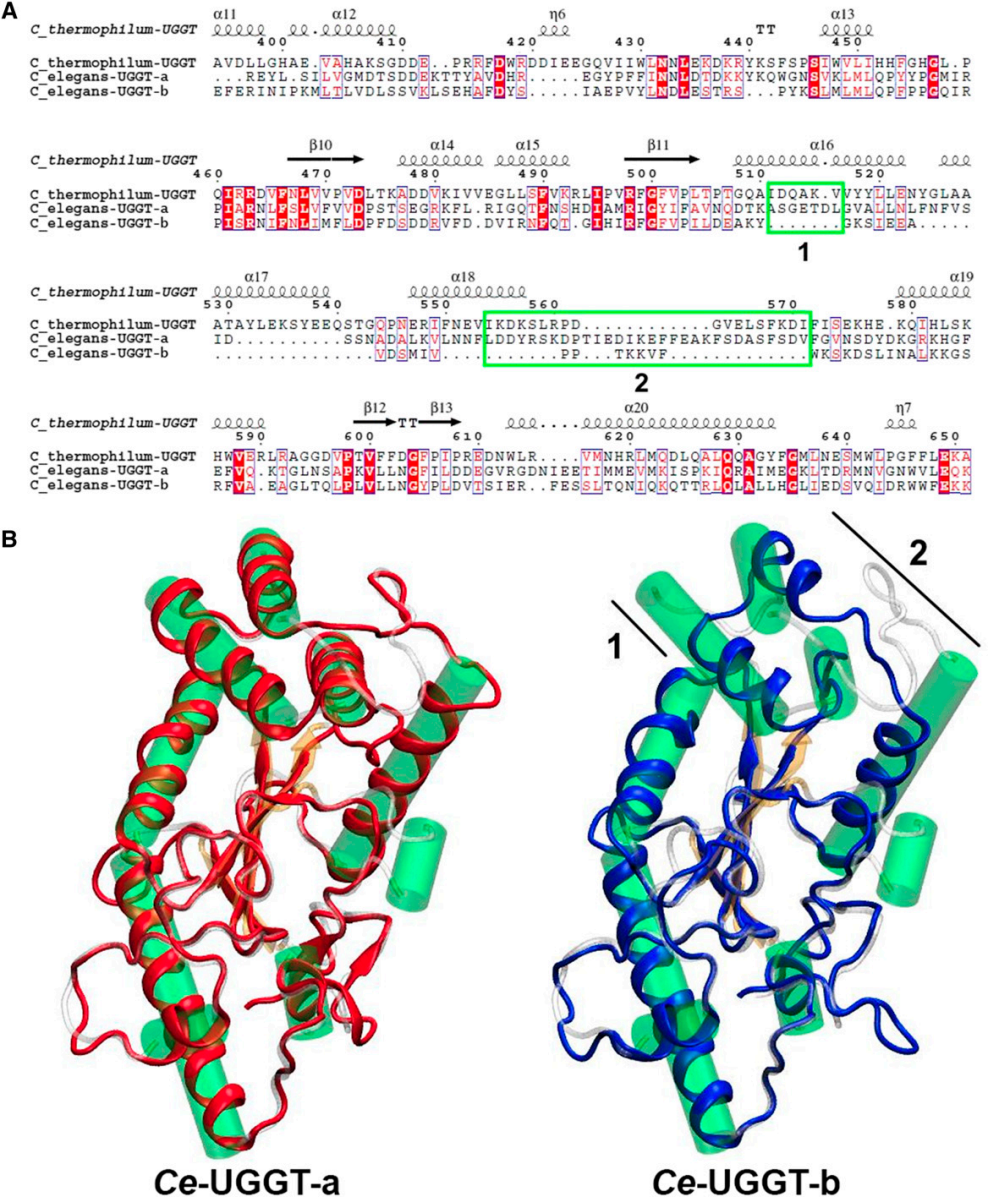
Sequence alignment of *Ce*-UGGT-a, *Ce*-UGGT-b, and UGGT of *Chaetomium thermophilum* TRXL2 domains (A) Green boxes denote missing regions (1 and 2) in UGGT-b sequence. Structural alignment of the *Ct*UGGT TRXL2 domain (PDBid 5NV4) with *Ce*-UGGT-a and *Ce*-UGGT-b (B) *Ce*-UGGT-a and *Ce*-UGGT-b are depicted in red and blue respectively and their structures are shown superimposed to *Ct*UGGT. Each region of *Ct*UGGT is colored according to their structures (alpha-helix, beta-sheet, and loops in green, yellow and white, respectively). Helices are represented by cylinders.

Thioredoxin-like2 (TRXL2) and thioredoxin-like3 (TRXL3) domains have the same fold belonging to ER luminal chaperones and they have previously been proposed to be involved in the recognition of misfolded proteins (Roversi *et al.* 2017). Global sequence alignment of UGGT-a, UGGT-b, and *Chaetonium thermophilus* TRLX2 domain shows that there are two small deletions (denoted as 1 and 2 in Figure 5A) in the TRLX2 domain of UGGT-b [30]. The structural models for TRLX2 domain of UGGT-a and UGGT-b show that the two missing regions are located in a highly solvent-exposed region, including part of an alpha helix and a large flexible loop (Figure 5B). These TRLX2 regions could be important for the recognition of misfolded proteins, and therefore their absence in UGGT-b results in the observed lack of UGGT activity.

**Figure 6:**
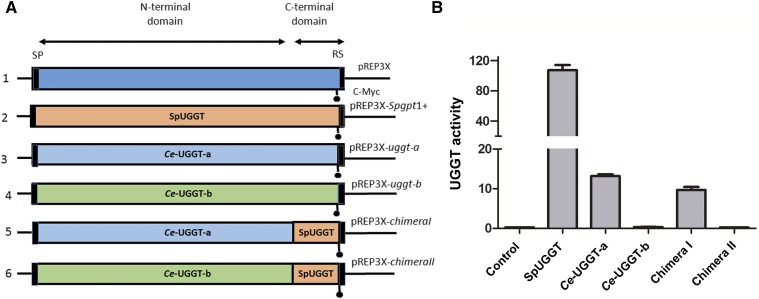
Structure of the expression plasmid encoding *Ce*-UGGT-a, *Ce*-UGGT-b and *Sp*UGGT (PANEL A) 1). Common structure of the constructions used in the UGGT activity assay. RS for retrieval signal, SP for signal peptide, C-*myc* indicates the insertion location of the c-Myc-encoding sequence. Expression plasmid encoding full-length SpUGGT, *Ce*-UGGT-a and *Ce*-UGGT-b, 2) 3) and 4, respectively and chimera I (*Ce*-N-term domain-UGGT-a fused to C-terminal SpUGGT) and chimera II (*Ce*-N-term domain-UGGT-b fused to C-terminal SpUGGT) 5) and 6) in that order. UGGT activity (Panel B) 0.3 mg of microsomal proteins obtained from *S. pombealg6 gpt1*- mutant cells transformed with expression vectors encoding full-length and chimeric UGGTs were incubated in a mixture that contained 5 mM Tris-HCl buffer, pH 7.5, 10 mM CaCl_2_ 0.6% Triton X-100, 5 mMNMDNJ and 3 µCi UDP-[14C]Glc at 24 °C for 30 min. Reactions were stopped with 1 mL of 10% of trichloroacetic acid. After centrifugation, the pellets were washed twice with 1mL of 10% trichloroacetic acid and counted. Activity values are expressed as is represented as counts 10^−3^ / minute /mg of microsomal protein. The values shown are the mean of three independent experiments. Error bar denotes standard deviations.

UGGTs are constituted by two highly structurally conserved domains and different chimeras have been constructed combining N-terminal and C-terminal domains from different UGGTs that produced fully active proteins (Arnold and Kaufman 2003; Takeda *et al.* 2016). In particular, Guerin and Parodi demonstrated that *D. melanogaster* and *S. pombe* UGGT N- and C-terminal domains were mutually replaceable by expressing chimeric proteins constituted by N-terminal domain of *D. melanogaster* UGGT fused to the *S. pombe* active C-terminal domain and the inverse construction in yeast (Guerin and Parodi 2003). To further analyze if UGGT-b N-terminal domain had lost its UGGT activity, c-*myc* labeled chimeric proteins constituted by the N-terminal *Ce*-UGGT-b domain and the *S. pombe* C-terminal catalytic domain, and by the N-terminal *Ce*-UGGT-a domain and the *S. pombe* C-terminal catalytic domain were expressed in mutant *alg6gpt1*- *S. pombe* cells together with the full-length c-*myc* labeled *Ce*-UGGT-a, *Ce*-UGGT-b, *Sp*UGGT (Figure 6A). All chimeric and full-length proteins were properly localized to the ER and expressed at comparable levels (Figure S3, Additional File 5). UGGT activity was assayed *in vitro* using yeast microsomes as an enzyme source (Figure 5B). As was previously found, *Ce*-UGGT-a was active although it displayed only 13% of that of SpUGGT in the same assay. *Ce*-UGGT-b was inactive as it has been previously reported (Buzzi *et al.* 2011). The chimeric protein constituted by the N-terminal domain of *Ce*-UGGT-a fused to the C-terminal domain of SpUGGT was active but showed a lower level of activity than *Ce*-UGGT-a (9% of SpUGGT activity) and the other chimeric protein constituted by the N-terminal domain of *Ce*-UGGT-b fused to the C-terminal domain of SpUGGT was fully inactive (less than 1% of SpUGGT activity).

Taken together the biochemical and the structural bioinformatics analysis support the idea that UGGT-b has lost its ability to recognize misfolded proteins and peptides but retained its glycosyltransferase activity.

## Discussion

The UDP-glucose:glycoprotein glucosyltransferase gene exists as a single orthologous gene in all major groups of eukaryotes. Only a few protists species that make either very short N-linked glycans or no N-linked glycans at all, lack the uggt gene (Banerjee *et al.* 2007; Samuelson and Robbins 2015). On the other hand, *Saccharomyces cerevisiae* genome encodes an inactive UGGT-like protein (Kre5p) that gained a new function (Castro *et al.* 1999). The only eukaryotic lineages that exhibit two copies of this gene are *Caenorhabditis* worms and vertebrates. In the present work, we aimed to gain insight into the origin and evolution of these paralogs and the emergence of a putative different function of *uggt-b* (different from *uggt-a*) in *Caenorhabditis*.

Bayesian phylogenetic inference based on UGGT protein sequences of an ample spectrum of eukaryotic species showed that *uggt* genes went through independent duplications in *Caenorhabditis* and vertebrates (Figure 1). The unique *uggt* genes of *Pristionchus*, *Oscheius*, *Diploscapter*, *Angiostrongylus*, *Dictyocaulus*, *Oesophagostomum* and *Strongylus* form a group that diverges as sister of the *uggt-a* genes (Figure 1), reflecting differences between genes and species tree. The sorting/extinction of one of the copies of uggt in the ancestor of these lineages could be the reason for this topology. Another possibility (that does not exclude the former) is that the phylogenetic signal between UGGT-a and UGGT-b was blurred because of the acceleration of the rate of substitution within the *uggt-b* genes. This acceleration is congruent with longer branches observed in the UGGT-b group (see Figure 1).

Vertebrates and *Caenorhabditis uggts* paralogous genes were retained throughout the diversification of these lineages: about 500 million years for vertebrates (Brazeau and Friedman 2015; Sugahara *et al.* 2016) and 50-80 million years for *Caenorhabditis*(Stein *et al.* 2003; Cutter 2008). Gene duplications had been proposed to be common events in vertebrates and *Caenorhabditis* but via different processes. Both whole-genome (Dehal and Boore 2005; Cañestro *et al.* 2013; Glasauer and Neuhauss 2014), and local duplications (Abbasi 2015) were proposed to occur in the ancestor and throughout the evolution of vertebrates providing the raw material for eventual evolutionary innovations and adaptations. Instead, only partial, inverted and chimeric –but no whole-genome –duplications were significant events in the genome of *Caenorhabditis* (Cavalcanti *et al.* 2003; Cutter *et al.* 2009). *Caenorhabditis uggt-b* gene is the result of a chimeric gene; it carries within its VII intron the coding sequence of a heat shock protein gene (*hsp70*) (“Wormbase”). *Caenorhabditis elegans* has fewer interchromosomal gene duplications than expected by chance (Semple and Wolfe 1999) so the location of *Ce-uggt-a* in the X sexual and *Ce-uggt-b* in an autosome could be considered an infrequent event.In *C. elegans*, *Ce-uggt-a* and *Ce-uggt-b* genes are located in the X (genetic position X:1.66) sexual chromosome and in the autosome 1 (genetic position 1:3,74), respectively (“Wormbase”). In *H. sapiens*, *HUGT1* and *HUGT2* genes are located in two autosomal chromosomes (the genetic position of *HUGT1* is 2q14.3 while that of *HUGT2* is 13q32.1)(“http://www.omim.org”). HUGT1, HUGT2, *Ce*-UGGT-a, and *Ce*-UGGT-b pairwise distance values were compared, with the lowest score belonging to human paralogs, a compatible result with a HUGT duplication posterior to the nematode-vertebrate common ancestor (NVCA) divergence (Figure 1). The highest value was between *Caenorhabditis* paralogs, reflecting that although their origin was posterior to the NVCA divergence (Figure 1), their evolution (at least in one of the two copies) has been accelerated.

Gene duplication constitutes a major source of novelty on which natural selection can occur. After duplication, the fate of a new gene copy might be pseudogenization by mutational decay (nonfunctionalization), acquisition of a new function (neofunctionalization) or preservation of both genes due to the complementary partitioning of the original function between the two duplicates (subfunctionalization) (Ohno 1999; Lynch and Force 2000; Prince and Pickett 2002). *Caenorhabditis uggt-b* and vertebrate *uggt-2* genes evolved in a background of purifying (negative) selection (Figures 3 and 4, see Results); nonfunctionalization of the duplicated gene did not take place in any of these lineages, reflecting that retention of these copies might have resulted advantageous, although maintenance does not exclude divergence.

The presence of two UGGT isoforms (HUGT1 and HUGT2) was first reported in humans (Arnold *et al.* 2000). HUGT1 displayed functional activity but HUGT2 did not, and for this reason, HUGT2 was believed to be an inactive homolog of HUGT1 (Arnold and Kaufman 2003). Nevertheless, a chimeric protein consisting of the non-catalytic portion of HUGT1 and the catalytic domain of human HUGT2 displayed glucosyltransferase activity, revealing that the carboxyl-terminal region of HUGT2 contains a catalytic domain that is functional and can replace that of HUGT1 (Arnold and Kaufman 2003). The inverse construction, a chimeric protein formed by the non-catalytic portion of HUGT2 and the catalytic domain of HUGT1 was inactive (Arnold and Kaufman 2003). Furthermore, using synthetic fluorescently labeled glycans and misfolded glycoproteins it was demonstrated that recombinant HUGT2 was enzymatically active and its glycan specificity was quite similar to that of HUGT1 (Takeda *et al.* 2014). Moreover, as mentioned, Takeda and collaborators confirmed that truncated proteins comprising only the C-terminal domain of both HUGT1 and HUGT2 were able to glucosylate synthetic substrates (Takeda *et al.* 2016). Although the catalytic activity of a recombinant HUGT2 seems to be rather low, these results raise the possibility that UGGT2 plays a similar role to UGGT1 in the ER of vertebrates (Takeda *et al.* 2014). Recently, the UGGT from a thermophilic yeast has been crystallized and structurally characterized, showing that UGGT has a novel seven-domain fold of complex topology (Roversi *et al.* 2017). This study suggests that efficient UGGT-mediated reglucosylation of misfolded glycoproteins of very different sizes and shapes depends on the conformational flexibility of an “interdomain” located between the C- catalytic terminal and the N-recognition terminal (Roversi *et al.* 2017). Different studies have shown that HUGT1 and HUGT2 proteins are widely expressed although with a different tissue and cellular expression pattern and a marked difference in their level of expression, being HUGT2 expression lower than that of HUGT1 in most analyzed tissues (“proteinatlas”; Arnold *et al.* 2000). On the other hand, some reports that indicate that both *HUGT*s are differentially regulated. *HUGT1* but not *HUGT2* is upregulated upon disruption of protein folding in the ER, while the expression of mouse *uggt* genes is differentially regulated by high concentrations of progesterone (Arnold *et al.* 2000; Prados *et al.* 2013). Moreover, increased expression levels of proteins associated with recognizing and modifying misfolded proteins, including HUGT2 and ER degradation-enhancing alpha-mannosidase-like protein 2 (EDEM2) but not HUGT1 were found in dorsolateral prefrontal brain cortex in schizophrenia (Kim *et al.* 2018). These results allow speculating that HUGT2 -and probably all vertebrate UGGT2- may be involved with a subset of specific client proteins that do not overlap with those recognized by HUGT1 -and all vertebrate UGGT1- resulting in the specialization of its function.

In vertebrates, the C-terminal domain of UGGT2 evolved under strong purifying selection, comparable to that found in UGGT1 (Table 3), leading to catalytic function conservation. Relaxation of purifying selection occurred in the recognition domain, although with lower intensity than in *Caenorhabditis* (Table 4). These observed mild levels of relaxation would not have affected non-native glycoprotein recognition function.

Patterns of molecular evolution analyzed in this work together with the mentioned differences in regulation and expression suggest that vertebrate UGGT2 underwent a specialization process, keeping UGGT activity but in a different cellular and developmental context. The occurrence of specialized paralogs has been reported in other proteins involved in the QC. The paralogs *crt* and *cnx* are examples of genes that have arisen from a common eukaryotic ancestor and gone through a specialization process (Banerjee *et al.* 2007). Both genes retained their functions; however, displaying differences in binding specificities. Furthermore, even though CNX and CRT share substrates, some proteins are exclusive clients of each one (Molinari *et al.* 2004; Lamriben *et al.* 2016). On the other hand, phylogenetic analyses suggest that *mns1*(=*ER mannosidase I*) and *edem* (= *ERAD-degradation-enhancing-α-mannosidase-like protein*) are also paralogs (Banerjee *et al.* 2007) and both display mannosidase activity; however, EDEM preferentially recognizes misfolded proteins (Shenkman *et al.* 2018).

*Caenorhabditis* UGGT-a and UGGT-b play different cellular and developmental roles(Buzzi *et al.* 2011). As mentioned above, *C. elegans*, *uggt-b* is an essential gene; homozygous *uggt-b* null mutants are not able to develop into progressive larval stages whereas *uggt-a* (*RNAi*) interfered worms have only minor phenotypes (Buzzi *et al.* 2011). These results show that *Ce*-UGGT-b is not functionally replaceable by a fully expressed and active *Ce*-UGGT-a in homozygous *uggt-b* mutants. Moreover, heterologous expression of *Ce-uggt-a* and *Ce-uggt-b* in *Schizosaccharomyces pombe* devoid of UGGT activity confirmed that *Ce*-UGGT-b did not display canonical UGGT activity although it was expressed at the same level as *Ce*-UGGT-a (Figure 6B and Figure S3) and (Buzzi *et al.* 2011). In addition, while *Ce-uggt-a* expression is regulated by the *ire-1* arm of the unfolded protein response pathway, *Ce-uggt-b* is not upregulated in response to the accumulation of misfolded proteins in the ER (Buzzi *et al.* 2011). A relevant clue that points to a different biological role (but one in which *Ce*-UGGT-b is still involved in alleviating ER stress) was provided by experiments of gene silencing by RNAi. When *ire-1uggt-b*(RNAi) mutant strain is treated with a very low tunicamycin concentration (that triggers a low level of accumulation of misfolded proteins), worms die or stop their development while no such effect was observed in *ire-1uggt-a*(RNAi). The lack of *Ce*-UGGT-b in early development causes general defects that produce larval arrest or cell death (Buzzi *et al.* 2011).Moreover, the levels of expression of *Ce-uggt*-a and *Ce-uggt*-b are completely different, being the former at least a hundred times higher than the latter during embryogenesis and development (see expression pattern for *C. elegans* *uggt*-1 and *uggt*-2 in Wormbase) (“Wormbase”). On the other hand, while *Ce*-UGGT-a is regularly expressed during the entire development, *Ce*-UGGT-b expression shows a minor peak only at 100 h after fertilization in the mesoderm and ectoderm tissues and in the transition between larva 2 to larva 3 stage (“Wormbase”).

It has been previously demonstrated that *Ce-uggt-b* homozygous deletion mutant strain is lethal (embryos are unable to develop or cannot progress to L2 stage, periods that coincide with the peak of expression of *Ce*-UGGT-b) and proposed that UGGT-b activity was not directly related to the QC cycle (Buzzi *et al.* 2011). The high levels of sequence conservation together with the low dN/dS ratio in the C-terminal domain of UGGT-b, suggest that this protein probably retained a glucosyltransferase function (Figures 2, 3, Table 2). Moreover, structural bioinformatics analysis and biochemical studies performed in this report pointed to the notion that UGGT-b has lost its canonical UGGT activity but has gained a new function retaining its glycosyltransferase activity but recognizing a new substrate (Figures 5 and 6).

Through their evolution GTs have diversified their activity by means of subtle structural changes that enriched the diversity of target substrates, such as lipids, proteins, nucleic acids and small organic molecules (Albesa-Jové and Guerin 2016). The recognition domain of *Caenorhabditis* UGGT-b depicted significant episodic diversifying selection. A shift in the type of substrate driven by mutation during redundancy may have favored the acquisition of a new biological role; that is, a process of neofunctionalization.

The maintenance of redundant functions of several duplicated genes in *C. elegans* was put in evidence by a combinatorial RNAi approach (Tischler *et al.* 2006). In most of these duplications, that occurred before the *C. elegan*s-*C. briggsae* divergence, 18 million years ago (ranging between 5-30 MYA) (Cutter 2008), both sequence and function were maintained by purifying selection in the two copies. However, this was not the case of *uggt* genes. Putative neofunctionalization of the *uggt*-b gene in *Caenorhabditis* seems to be counterintuitive to the claimed maintenance of gene functions -particularly those controlling development- among model organisms as *C. elegans*. Even more, the divergent evolutionary fates of *uggt-2* and *uggt-b* genes in the only two eukaryotic lineages that carry these duplicated genes highlight the need to take precautions before generalizing gene functions in model organisms.

## Conclusions

Two independent duplications originated the second *uggt* copy in *Caenorhabditis* and vertebrates lineages, giving rise to different evolutionary pathways of the resulting copies. In vertebrates, UGGT2 may interact with a subset of specific client proteins with minimum or null overlap with those recognized by UGGT1, resulting in the specialization of its function in a background of purifying selection. Within *Caenorhabditis*, UGGT-b-as evidenced by its sequence evolution and functional assays- has acquired a new role that remains to be characterized, depicting a neofunctionalization process. The independent origin and divergent functions of *uggt-2* and *uggt-b* in both lineages should alert about the improperness of treating them as orthologs.
